# Single Molecule Characterization of Amyloid Oligomers

**DOI:** 10.3390/molecules26040948

**Published:** 2021-02-11

**Authors:** Jie Yang, Sarah Perrett, Si Wu

**Affiliations:** 1 National Laboratory of Biomacromolecules, CAS Center for Excellence in Biomacromolecules, Institute of Biophysics, Chinese Academy of Sciences, 15 Datun Road, Chaoyang District, Beijing 100101, China; jie.yang.jy546@yale.edu (J.Y.); sarah.perrett@cantab.net (S.P.); 2 Department of Cell Biology, Yale School of Medicine, New Haven, CT 06520, USA; 3 University of the Chinese Academy of Sciences, 19A Yuquan Road, Shijingshan District, Beijing 100049, China

**Keywords:** single molecule fluorescence detection, amyloid oligomers, protein aggregation, neurodegenerative disease

## Abstract

The misfolding and aggregation of polypeptide chains into β-sheet-rich amyloid fibrils is associated with a wide range of neurodegenerative diseases. Growing evidence indicates that the oligomeric intermediates populated in the early stages of amyloid formation rather than the mature fibrils are responsible for the cytotoxicity and pathology and are potentially therapeutic targets. However, due to the low-populated, transient, and heterogeneous nature of amyloid oligomers, they are hard to characterize by conventional bulk methods. The development of single molecule approaches provides a powerful toolkit for investigating these oligomeric intermediates as well as the complex process of amyloid aggregation at molecular resolution. In this review, we present an overview of recent progress in characterizing the oligomerization of amyloid proteins by single molecule fluorescence techniques, including single-molecule Förster resonance energy transfer (smFRET), fluorescence correlation spectroscopy (FCS), single-molecule photobleaching and super-resolution optical imaging. We discuss how these techniques have been applied to investigate the different aspects of amyloid oligomers and facilitate understanding of the mechanism of amyloid aggregation.

## 1. Introduction

Cellular proteostasis is one of the key problems in biology and has been explored for decades. Within the complex cellular environment, newly synthesized polypeptides may be prone to misfold under certain conditions. Failing to eliminate misfolded proteins results in their accumulation and self-assembly into a variety of aggregates, such as the β-rich fibrillar structures termed amyloid [1,2,3]. The misfolding and aberrant aggregation of proteins and peptides into amyloid fibrils is associated with many human diseases, including the most common neurodegenerative diseases Alzheimer’s and Parkinson’s as well as type 2 diabetes [4,5]. In addition, the ability to form amyloid is found to be a common or generic property of polypeptide molecules and amyloid structures execute special biological functions in a range of cellular processes in living organisms [6,7]. Amyloid aggregation is a complicated process involving the transition from soluble monomers to insoluble aggregates. Although great effort and progress has been made to reveal the structures and assembly mechanisms of the β-sheet-rich amyloid fibrils [8,9,10,11,12], much evidence indicates that the oligomeric intermediates formed during the amyloid fibril formation process rather than mature fibrils are toxic to cells and are the major pathogenic agent in neurodegenerative disease [13,14,15,16]. Moreover, these oligomeric species with a wide range of sizes and distinct conformations are also crucial intermediates of amyloid aggregation. Therefore amyloid oligomers have attracted great interest during recent years and are considered to be potential targets for the development of therapeutic strategies [17].

It is desirable to study the structure, quantity and properties of oligomers during the process of amyloid aggregation. However, due to the low-populated, metastable and transient nature of amyloid oligomers, only limited information can be obtained using conventional bulk techniques such as the thioflavin T (ThT) fluorescence assay, circular dichroism spectroscopy (CD), dynamic light scattering (DLS), electron microscopy (EM) and nuclear magnetic resonance (NMR) spectroscopy, given that the oligomeric species are vastly outnumbered by monomeric and larger aggregate populations [18,19,20,21,22,23]. A useful strategy is to stabilize the amyloid oligomers in some way in order to obtain homogeneous samples, such as by lyophilization [24,25,26], incubation with chemicals [25,27], or preparation in membrane-mimicking environments [26,28,29,30,31] which then makes it possible to separate out oligomers and subject them to structural and functional studies. However, this approach does not allow real-time observation of the dynamic changes that occur during conversion of monomers to oligomers. Furthermore, the stabilization process may perturb the structure and properties of oligomers making them different from those that form under native conditions in solution during aggregation. In contrast, fluorescence microscopy-based single molecule techniques have advantages over conventional biochemical and biophysical methods and can characterize individual protein molecules, allowing exploration of the highly heterogeneous and dynamic amyloid oligomers. By obtaining information at the single molecule level, species that represent only a small proportion of the system can nevertheless be detected, instead of being hidden and averaged as in the case of ensemble experiments, thus providing insight into the process of amyloid aggregation in unprecedented detail.

There are two primary experimental configurations for single molecule fluorescence detection, namely confocal microscopy and total internal reflection fluorescence (TIRF) microscopy. In the confocal setup, a collimated laser beam is focused by a high numerical aperture objective onto a diffraction-limited femtoliter volume. Fluorescence-labeled molecules at picomolar concentration in solution can then be excited to give fluorescence bursts when diffusing across the focal volume. In the TIRF setup, the laser is reflected by the bottom of the coverslip and the evanescent wave excites the fluorescence-labeled molecules that are immobilized or adsorbed on the surface of the coverslip. In contrast to the short observation time in the solution confocal setup (<1 ms), the surface-immobilized fluorescent molecules can be observed for longer time periods (seconds to minutes) before photobleaching. Under both setups, single-molecule Förster resonance energy transfer (smFRET) can be performed, in which a protein of interest is site-specifically labeled with a donor-acceptor dye pair and the distance change between the labeling sites is represented by the FRET efficiency. For most commercial dyes, FRET efficiency is sensitively dependent on the donor-acceptor distance in the range of 2–10 nm, thus is particularly suitable for investigating protein folding, structural transitions, and the populations and dynamics of specific protein conformations [32,33]. The confocal setup can also be employed for fluorescence correlation spectroscopy (FCS) which is based on the fluorescence fluctuations originating from the fluorescent molecules diffusing across the focal volume [34,35]. By analyzing the auto-correlation curves of fluorescence fluctuation, quantitative information such as the concentration, diffusional coefficient and dynamic properties of molecules can be obtained [36]. SmFRET and FCS have been applied to study the conformations and dynamics of amyloidogenic proteins including the Alzheimer’s-related amyloid β-peptide (Aβ) [37] and Tau [38,39,40], the Parkinson’s-related protein α-synuclein [41,42,43,44,45,46], the amyotrophic lateral sclerosis-related protein TAR DNA-binding protein 43 (TDP-43) [47], Huntington’s disease-related huntingtin [48], and the yeast prion proteins Ure2 [49] and Sup35 [50]. Moreover, the effects of surfactants, aggregation inducers, interacting partners, post-translational modifications and disease-related mutations on the conformations and folding of these amyloidogenic proteins have also been extensively analyzed in the studies listed above. These studies provide insight into the intramolecular conformational ensemble of amyloid proteins in both native and aggregation-prone states and facilitate the understanding of their conversion from soluble protein to insoluble aggregates. Since this work has already been reviewed elsewhere [51,52,53], in this review, we will mainly focus on current progress in the detection of intermolecular oligomers at the single molecule level, the characterization of their structural and dynamic properties, their generation and depletion kinetics, and how their properties are associated with cytotoxicity and their regulation by different factors.

## 2. Characterization of Amyloid Oligomers by Single Molecule Fluorescence Detection

In order to detect the oligomeric species formed during amyloid fibrillization by smFRET, monomers are labeled with donor and acceptor fluorophores, mixed in equal amounts and then incubated to initiate aggregation. The formation of oligomers gives FRET signals that can be detected as coincident bursts in donor and acceptor detection channels when the oligomers diffuse across the focus even in the presence of an excess of monomers [54,55,56,57]. Two-color coincidence detection (TCCD) is an alternative detection mode, which similarly to smFRET is based on a confocal setup but is particularly suitable for samples with low intermolecular FRET efficiency. For TCCD the protein molecules are labeled with two spectrally separated fluorophores that are excited by an overlapped dual-color laser beam [58]. The fluorescence bursts occurring in only one emission channel are counted as monomers, while simultaneous fluorescence emission in both channels is counted as oligomers [59]. The oligomer concentration can be determined by converting the oligomer proportion referenced to a standard sample with known concentration. It is also possible to obtain information about the size and conformation of oligomers by analyzing the fluorescence intensity and FRET efficiency of oligomer bursts. During single molecule experiments, samples need to be diluted to the picomolar concentration range which may cause dissociation of unstable oligomeric species. In order to avoid this, fast-flow microfluidic techniques can be combined with single molecule detection, by which the acquisition time can be shortened from hours to minutes and so reduce the opportunity for oligomers to dissociate after dilution [60,61].

Amyloid oligomers can also be studied by TIRF imaging using either a single-color laser or coupled with TCCD. The samples are deposited onto the coverslip and excited by single-color or two overlapped lasers of different wavelengths. The intensity and colocalization of fluorescence in the images can be analyzed to give detailed information about the composition, size and morphology of the oligomeric species present [62]. An alternative to covalently labeling the target proteins with organic fluorophores, is to directly observe the unlabeled amyloid aggregates by TIRF imaging in the presence of a structure-specific dye such as ThT or pentameric formyl thiophene acetic acid (pFTAA) [63,64,65,66]. This can avoid exogenous effects introduced by covalently-linked dyes and photobleaching in conventional imaging. However, the resolution of traditional far-field optical imaging is around 250 nm due to the optical diffraction limit, far beyond the scale of amyloid oligomers and aggregates which are usually in the range from a few nanometers up to tens of nanometers. The development of super-resolution imaging techniques, either based on stochastic optical reconstruction microscopy (STORM) or stimulated emission depletion microscopy (STED), has now greatly improved the optical resolution to below 20 nm, allowing the exploration of the morphology and properties of amyloid aggregates on the nanoscale [67,68,69,70,71].

### 2.1. The Size Distribution of Amyloid Oligomers

**Figure 1:**
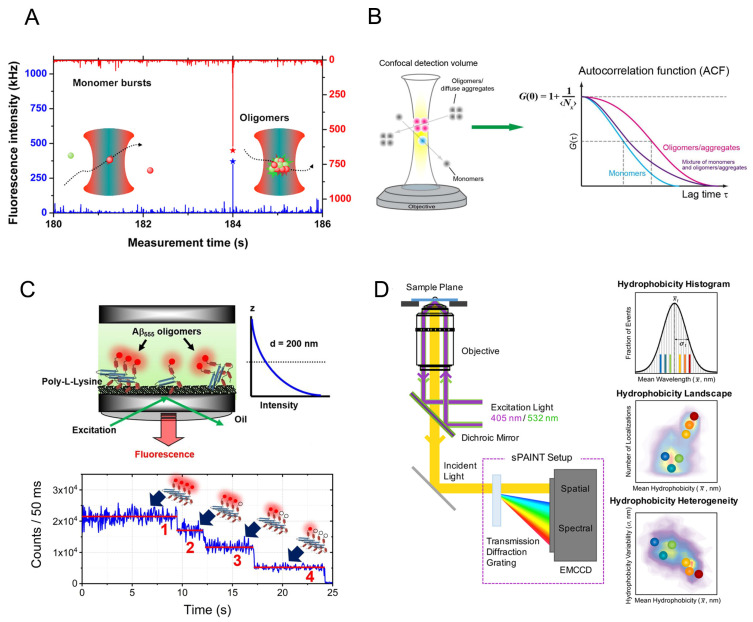
Overview of single molecule fluorescence methods for detecting amyloid oligomers. (**A**) Confocal two-color coincidence detection (TCCD) of amyloid oligomers. Formation of oligomers by donor and acceptor labeled monomers can generates coincident bursts in both detection channels when diffusing across the focal volume. Figure reproduced from ref. [59] with permission. (Copyright (2008) National Academy of Sciences.) (**B**) Fluorescence correlation spectroscopy (FCS) measurements of fluorescence-labeled monomers and oligomers which show different diffusion times (DT) in the autocorrelation curves. Figure adapted from ref. [36] with permission. (**C**) Single molecule total internal reflection fluorescence (TIRF) imaging of surface-immobilized amyloid oligomers. The fluorescent sample is excited by the exponentially decaying evanescent field at the coverslip-sample interface. The single molecule photobleaching steps can be counted to determine the oligomer size. Figure adapted from ref. [73] with permission. (**D**) Single molecule spectrally-resolved points accumulation for imaging in nanoscale topography (sPAINT) setup for super-resolution imaging of individual amyloid oligomers as well as the surface hydrophobicity properties. Figure reproduced from ref. [68] with permission. (Further permissions related to this figure should be directed to the ACS. Original figures at: https://pubs.acs.org/doi/10.1021/acs.nanolett.8b02916, accessed on 29 December 2020).

The Klenerman group has pioneered the development of single molecule detection of amyloid oligomers [54,55,56,59,62]. Orte et al. [59] presented the first application of TCCD to study the amyloid oligomer formation of the SH3 domain of phosphatidylinositol-3′-kinase (PI3-SH3) (Figure 1A). Two kinds of oligomer with different stability upon dilution were detected. The apparent size of the oligomers could be estimated by comparing the fluorescence burst intensity of oligomers to monomers. The early oligomeric species were found to be the most cytotoxic with an average size of 38 ± 10 monomers, which remained constant throughout the aggregation reaction. The stability of PI3-SH3 oligomers increases substantially with time, suggesting a conformational change from early to late oligomers. Analysis of oligomer size based on burst intensity has been applied in subsequent confocal smFRET studies [54,55]. However, due to the different paths taken by the oligomers as they diffuse or transfer across the focal volume of the instrument, as well as the presence of fluorescence quenching within the oligomers, a precise oligomer size cannot be obtained using this method, although the quenching can be corrected using an advanced multi-dimensional smFRET measurement [72]. Therefore, the obtained apparent oligomer size can be considered to be a qualitative indication when comparing the relative sizes of oligomers at different time points or for different mutants.

Since FCS can measure the diffusion coefficient and hydrodynamic radius of fluorescently labeled molecules, it can be employed to detect the formation of high molecular weight species even in an inhomogeneous system (Figure 1B). By FCS, the early oligomer formed by fluorescent-labeled α-synuclein in the presence of excess unlabeled α-synuclein was identified, and the distribution of diffusion coefficients showed a transient intermediate peak attributed to the dimer, as well as a broader population corresponding to higher oligomers [74]. The effect of solvents such as SDS, urea, glycerol, as well as nanoparticles on the oligomerization of α-synuclein and its mutants was also studied by measuring diffusion coefficient using FCS [75,76]. In order to identify a small subpopulation of oligomers without the influence from the monomer signals, a combination of FRET and FCS can be applied, where only the fluorescence in the acceptor channel generated by oligomers is subjected to FCS analysis [75,77]. Using FRET-FCS, the size of Aβ42 oligomers formed at physiological concentrations in solution was measured to be 11 ± 3 monomers, which represents only as low as a 2% fraction of the total amount of monomers [77]. In most FCS studies, the target proteins need to be covalently labeled with fluorophores, which may have effects on amyloid oligomerization. A strategy termed probe enhancement FCS (PE-FCS), in which the amyloid-binding probe undergoes fluorescence enhancement when bound to amyloid aggregates compared to the free form in solution, was developed to directly monitor the oligomerization of unlabeled amyloidogenic proteins without covalent modification [78]. Using such a probe, the early assembly intermediates of Aβ were detected, which are 64–82 nm in size. This approach is potentially applicable to detect the formation and size distribution of amyloid species in human biofluids such as cerebrospinal fluid and blood serum [79].

The size distribution of amyloid oligomers can also be determined by single-molecule photobleaching under confocal or TIRF imaging (Figure 1C) [73]. When fluorescent labeled monomers within the oligomer are illuminated, the fluorophores bleach one at a time, creating a stepwise intensity-versus-time trajectory. By counting the photobleaching steps, the number of monomers contained in an oligomer can be determined. Using single-molecule photobleaching techniques, the Gafni/Steel group studied Aβ oligomer assembly and size distribution at physiological concentrations in solution, on the surface of model lipid membranes and neuronal cell membranes [80,81,82,83,84]. It was found that at low concentration near physiological conditions, the major size population of Aβ oligomers in solution is mainly in the dimer-to-tetramer range, while the membrane facilitates the formation of surface-bound oligomers, leading to a significantly larger size distribution in comparison to oligomers formed in solution [81]. In a recent study of Aβ40 and Aβ42 on a supported lipid bilayer at nanomolar concentration, Aβ monomers are found to be tightly associated with the membrane and are highly mobile whereas trimers and higher-order oligomers are largely immobile. Interestingly, the oligomer growth of Aβ40 on the membrane is more rapid than the more amyloidogenic Aβ42, and is inhibited by a 1:1 Aβ40/Aβ42 mixture [83]. The oligomer size distribution of islet amyloid polypeptide (IAPP) oligomers was also measured by single-molecule photobleaching and their membrane affinity was analyzed, which shows that oligomers (dimers, trimers and tetramers) have much higher membrane affinity than monomers [85]. These studies broaden knowledge of the heterogeneity of oligomer size and the interaction between oligomers and membranes at physiological concentrations, which sheds light on the mechanisms of cytotoxicity of amyloid oligomers.

The limitation of the single-molecule photobleaching technique is that if the oligomers consist of a large number of monomers, it is hard to accurately determine the number of photobleaching steps from the exponential decay of the fluorescence intensity curve. Additionally, the presence of multiple fluorescent labels may lead to fluorescence quenching and may also influence the aggregation process of the protein. To solve these problems, Zijlstra et al. [86] applied single-molecule photobleaching in combination with a sub-stoichiometric labeling strategy, in which only a fraction of the monomers contain fluorescent labels. During the aggregation process, the incorporation of labeled monomers in the oligomer is a stochastic process, therefore, it becomes possible to predict the label probability mass function and link the number of fluorescent labels to the total number of monomers. Based on this method, two distinct species of α-synuclein oligomers with sizes of 15–19 monomers and 34–38 monomers were detected by analyzing the bleaching steps of sub-stoichiometric labels [87].

### 2.2. Conformational Diversity of Amyloid Oligomers

The toxicity of amyloid oligomers is not only related to their size but also to their conformation and their surface properties [88,89]. While super-resolution imaging can provide localization information for fluorescent molecules at nanoscale precision, combination of this technique with the simultaneous detection of other fluorescence parameters can reveal multi-dimensional properties of the fluorescence-labeled proteins (Figure 1D). For example, a spectrally-resolved super-resolution imaging method termed sPAINT (spectrally-resolved points accumulation for imaging in nanoscale topography), takes advantage of Nile red dye that can transiently bind to amyloid aggregates and sensitively reflect their hydrophobicity according to the wavelength of the fluorescence emission, allowing the morphology and surface hydrophobicity of the individual aggregates of α-synuclein and Aβ42 to be detected simultaneously at super-resolution [67,68]. The sPAINT images show that the oligomeric species of α-synuclein have higher hydrophobicity than the fibrillar species, which correlates with cytotoxicity. Furthermore, by combining sPAINT with ThT-stained imaging, the oligomers containing β-sheet structure formed during the aggregation of α-synuclein could be further distinguished. The surface hydrophobicity of the ThT-inactive species shows little change during the aggregation reaction, whereas the ThT-active species show a decrease in hydrophobicity [68]. This approach has also been applied to characterize the structure and surface hydrophobicity of the prion protein, PrP. Based on the ThT intensity, the surface hydrophobicity and the proteinase K resistance, at least five types of oligomers were identified during PrP aggregation, suggesting structural conversion from proteinase K-sensitive oligomers to proteinase K-resistant oligomers [69]. The above studies indicate that the surface properties of aggregates change with time and amyloid oligomers are structurally diverse and distinct from fibrils.

Since the FRET efficiency is inversely related to the sixth power of the distance between the fluorescence labels, single-molecule FRET is a sensitive method to explore the structures of oligomeric assemblies. Oligomers with distinct conformations and structural compactness, can be distinguished using the FRET efficiencies values. Cremades et al. [54] used smFRET to monitor time evolution of oligomers during the aggregation process and identified two types of oligomers with different FRET efficiencies that emerge at different stages. The populations of α-synuclein oligomeric species were characterized as a function of their FRET efficiency and apparent oligomer size, in which the distribution of FRET values appears to vary with the size of the oligomers, revealing two dominant populations, one corresponding to a smaller size with lower FRET values (0.4–0.7) and the other corresponding to larger species with higher FRET values (0.6–0.9). The early emerging lower FRET species correlate with the loose assembly that contains less cross-β structure and is protease-K sensitive. The higher FRET species correlate with the tight β-sheet stacking which is proteinase-K-resistant and shows high cytotoxicity. Conformational conversion from early low-FRET oligomers to late high-FRET oligomers was suggested to occur [54]. Base on this observation, the conformational properties of the α-synuclein oligomers of pathological mutants, A53T, A30P and E46K, were characterized [90]. Whilst the total concentration of oligomers generated by these mutants is comparable to wild type (WT) during the lag phase of aggregation, the mutants show different FRET efficiency distributions, suggesting that the conformational properties of oligomers may be more relevant to cytotoxicity than the absolute concentration of oligomers [90].

**Figure 2:**
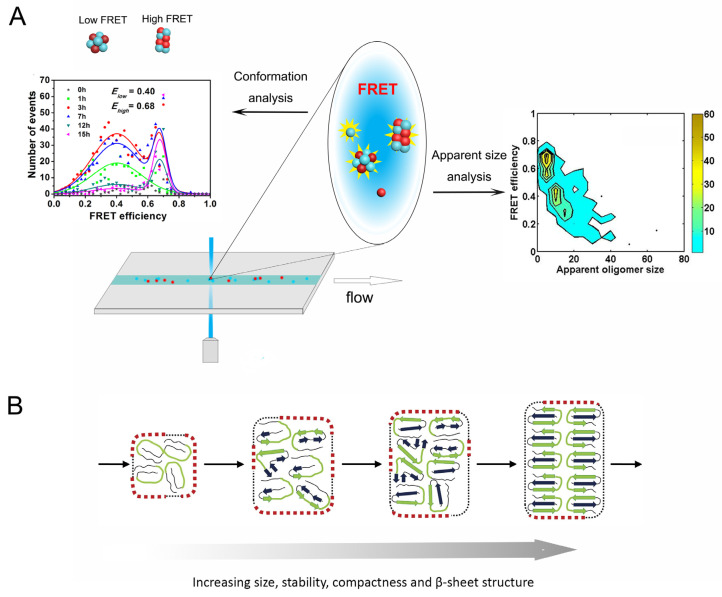
Different conformations of amyloid oligomers detected by confocal single molecule Förster resonance energy transfer (smFRET). (**A**) The FRET distribution of amyloid oligomers at different time points show low- and high-FRET populations which have different apparent sizes. Part of figure reproduced from ref. [57] with permission. (Further permissions related to this part of the figure should be directed to the ACS. Original figures at: https://pubs.acs.org/doi/10.1021/jacs.7b10439, accessed on 29 December 2020). (**B**) Schematic representation of the conformational conversion of amyloid oligomers during aggregation reaction. Figure adapted from ref. [15] with permission.

In recent work by our group, oligomers with different conformations formed during amyloid aggregation were detected by smFRET for the yeast prion protein Ure2 [57]. We found that the initial low-FRET oligomers of Ure2 are metastable and probably disordered, while the later high-FRET oligomers are more compact, showing similar FRET efficiency to oligomers dissociated from mature fibrils (Figure 2A). Therefore, a conversion step from the initial disordered oligomers into structurally compact oligomers is probably required, in order for compact oligomers in turn to be converted into growing fibrils. The oligomer conversion rate was determined to be slow and is the rate-limiting step in amyloid nucleation [54,57], thus the kinetic barrier for generating elongation-competent oligomers is high.

The conformational reorganization between early disordered oligomers and subsequently formed β-sheet-rich oligomers during amyloid formation has also been suggested for other amyloidogenic proteins such as the SH3 domain of α-spectrin [72,91], Aβ40 [92] as well as the yeast prion protein Sup35 [50], based on smFRET or other experiment techniques; this may be a general mechanism for generating elongation-competent amyloid nuclei (Figure 2B). As indicated by simulations and theoretical work, hydrophobic interactions play a crucial role in the formation of the initial disordered oligomers, while the intra- and intermolecular hydrogen bonding within β-sheets is considered to be the driving force for the subsequent conformational conversion [93,94]. Formation of hydrogen bonds compensates for the disruption of hydrophobic interactions in the initial disordered oligomers, thus favoring conformational reorganization to β-sheet structure to achieve the lowest energy state.

### 2.3. Kinetics and Dynamics of Oligomerization during Amyloid Aggregation

Kinetic modeling is being developed as a powerful tool to reveal the fundamental microscopic reaction steps during the amyloid aggregation process [95,96,97,98,99,100]. The fundamental theoretical work done by Knowles et al. [95] provided an analytical solution to the kinetics of fibril formation. By global fitting of a series of fibril formation curves over a range of concentrations obtained by bulk ThT assay, the coupled kinetic parameters *k*_n_*k*_+_, *k*_+_*k*_−_, and *k*_+_*k*_2_ can be obtained, where *k*_n_, *k*_+_, *k*_−_ and *k*_2_ represent the primary nucleation, fibril elongation, fragmentation, and secondary nucleation rates, respectively [97,98,100]. However, the primary nucleation process in the model is coarse-grained and simplified because of a lack of experimental data regarding the microscopic reaction steps of amyloid nuclei formation. Thanks to the advent of the single molecule fluorescence techniques discussed above, characterization of the time evolution of oligomeric intermediate species present during amyloid fibril formation becomes possible. In kinetic experiments, aliquots of fluorescently labeled samples are taken from the solution at different incubation times and subjected to smFRET detection. The oligomerization data obtained by smFRET and the fibrillization data obtained by ThT assay can be globally described with a more explicit kinetic model. Combined smFRET and kinetic analysis facilitates a quantitative understanding of the microscopic reaction mechanism of amyloid aggregation and the population changes of toxic oligomers during this process [54,55,56,57].

Using smFRET, Shammas et al. [55] studied the oligomerization of the K18 truncation of Tau and its mutants, ΔK280 and P301L, which are linked with familial frontotemporal dementia. The formation of K18 oligomers precedes fibril formation. They built a nucleation-conversion-polymerization model in which protein aggregation proceeds via monomer assembly into small oligomers, followed by slow structural conversion into fibrillar species. In contrast to the nucleation-polymerization model [95,96] which treats the oligomers as small fibrils and fails to describe the experimental data in this case, this model considers the oligomers to be structurally distinct from small fibrils and well describes the oligomerization and fibrillization curves globally, allowing it to be quantitatively determined how the mutations alter the aggregation pathway. Using the obtained kinetic parameters, the rate of spreading of oligomeric seeds in the cellular environment can be estimated [55,101]. However, in this study, the FRET distribution histograms of the K18 oligomers do not show different populations but only a broad distribution. In a later study, by changing the dilution buffer to different ionic strength conditions, two distinct classes of K18 oligomers with different stability during the aggregation reaction have been resolved, of which the first type is in rapid exchange with monomers and the second type is kinetically more stable and is probably off-pathway, indicating the oligomer diversity of Tau during the aggregation reaction [102].

In early single-molecule studies of α-synuclein, its aggregation was described with a simple conversion model [54], which indicated that conformational conversion from the initial oligomers to more compact oligomers is the rate-limiting step. Iljina et al. [56] extended the smFRET detection of the kinetics of α-synuclein oligomerization to a wide range of initial concentrations, from 0.5 μM to 140 μM. A kinetic model was developed to globally fit the changes in monomer decrease and oligomer increase data to determine the rate constants for the individual steps of the reaction [56]. They found that the amyloid formation of α-synuclein involves two unimolecular structural conversion steps, from the initial low-FRET oligomers to more compact high-FRET oligomers and then to fibrils, the latter of which elongate by monomer addition. The concentrations and numbers of aggregates necessary for effective seeding of α-synuclein were assessed according to the kinetic model at physiologically relevant conditions. The simulation shows that a high concentration of aggregates is needed for effective seeding while quantitative cellular assays reveal that cytotoxicity occurs at dramatically lower oligomer concentrations. It is likely that the spreading of α-synuclein under in vivo conditions involves not only templated seeding but also oligomer-induced cellular stress processes that amplify the aggregation [56].

**Figure 3:**
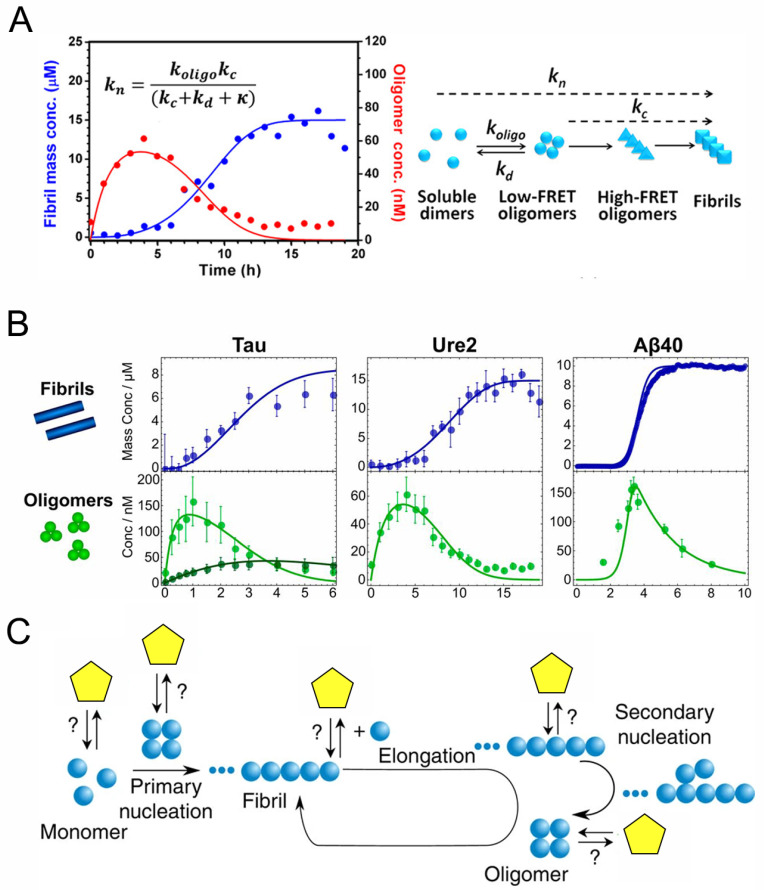
Kinetic analysis of amyloid aggregation. (**A**) A detailed kinetic model considering oligomer formation, dissociation, conformational conversion, fibril elongation and fragmentation is applied to describe the oligomerization measured by smFRET (red) and fibrillization measured by ThT (blue) globally. Figure reproduced from ref. [57] with permission. (Further permissions related to this part of the figure should be directed to the ACS. Original figures at: https://pubs.acs.org/doi/10.1021/jacs.7b10439, accessed on 29 December 2020). (**B**) Global fitting of ThT (blue) and smFRET (green) data from different amyloid aggregation systems. Figure reproduced from ref. [105] with permission. (**C**) The possible effects of different factors (yellow symbols) on the microscopic mechanisms during amyloid aggregation. Figure adapted from ref. [106] with permission.

The above studies using combined smFRET and kinetic analysis have greatly deepened understanding of oligomer generation, conversion and the quantitative relationship between oligomer populations and the amyloid aggregation process. However, the kinetic analysis in a number of previous studies has either focused only on the early stages of oligomer formation [54,69,72], or continued to use a coarse-grained nucleated polymerization model in which secondary processes were not considered [55]. Recently, our group studied the oligomerization mechanism of the yeast prion protein Ure2 [57], as mentioned above. The oligomerization and fibrillization of Ure2 was monitored by smFRET and ThT assay respectively under the same incubation conditions. Two kinds of Ure2 oligomers with different FRET efficiency distributions and emerging at different stages were observed which is similar to α-synuclein [54,56]. Since the fibril formation of Ure2 is fragmentation-dominated [99], we established an oligomerization/dissociation-conversion-elongation-fragmentation model to globally describe the ThT and smFRET data, taking into account the fibril fragmentation process. The coarse-grained primary nucleation rate was explicitly derived based on oligomer formation, dissociation and conversion as well as fibril growth and fragmentation (Figure 3A). The most important finding of this study is that the majority of the initial prefibrillar oligomers are metastable and dissociate rather than convert into fibrils. Only a small proportion of such initial oligomers can slowly convert into structurally compact oligomers, which in turn convert into growing fibrils. Moreover, the kinetic analysis also reveals that fragmentation is responsible for the autocatalytic self-replication of Ure2 fibrils, in contrast to that observed for peptides and proteins associated with neurodegenerative disease, such as Aβ [98], α-synuclein [103] and IAPP [104], of which surface-catalyzed oligomer generation occurs at a significant rate. This work provides insight into why functional amyloid systems are not toxic to their host organisms.

In a subsequent study, Dear et al. [105] developed a general chemical kinetics framework to investigate the properties of amyloid oligomers, which is the first systematic work on the oligomerization mechanism of different types of amyloid proteins (Figure 3B). By fitting the kinetic data of several unrelated amyloidogenic proteins obtained by either smFRET or other techniques [56,57,102,107,108], three key parameters that describe the dynamic properties of oligomers, i.e., half-life, productivity, and abundance, were defined and determined. Although the oligomers show remarkably different kinetic and thermodynamic stabilities, a common feature has been found: most of the oligomers are non-fibrillar and will dissociate back to monomers rather than mature into fibrillar species [105]. It was also shown that the oligomers of disease-associated amyloid proteins (Aβ, α-synuclein and Tau) have high abundance while oligomers from functional amyloid (Ure2) exhibit low abundance, providing hints as to the basis for the differences in toxicity between amyloid proteins.

### 2.4. Thermodynamic Properties of Amyloid Oligomers

Under physiological conditions, isoforms or mutational variants of amyloidogenic proteins may exist together with the WT to form co-aggregates [109]. For instance, data from in vivo and in vitro studies has established the interaction and co-oligomerization of two major Aβ isoforms, Aβ40 and Aβ42 [110,111,112]. It is essential to determine the propensity of formation of self-oligomers and co-oligomers, as well as the stability of the resulting oligomers, in order to assess the relationship between oligomeric properties and toxicity. However, given the low population of oligomers present under physiologically relevant conditions, it is not possible to use bulk methods to perform such thermodynamic measurements. Using single molecule fluorescence detection in combination with statistical mechanical modeling, the co-oligomerization of Aβ40 and Aβ42 was studied in the nanomolar range [113]. The key parameter describing the oligomerization process is the Gibbs free energy for monomer addition to an oligomer or another monomer, ΔG°, independent of oligomer size. By analyzing the equilibrium oligomer concentration data obtained by TCCD, it has been found that at low concentrations, the free energy penalty for forming co-oligomers is small, suggesting that the coexistence of self- and co-oligomers is possible and may potentially play a significant role in Alzheimer’s disease. This model can be used to simulate the self- and co-oligomer formation of Aβ isoforms at physiological concentrations at different isoform ratios. An increasing Aβ42/Aβ40 ratio is suggested to influence the fraction of oligomers containing Aβ42 as well as the hydrophobicity of the oligomers, thus increasing the extent of binding to cell membranes and the cytotoxicity.

The phenomenon of co-oligomerization of α-synuclein with its mutants as well as other amyloidogenic proteins has also been reported [114,115,116]. The equilibrium populations of oligomers formed by WT α-synuclein with familial A30P, A53T, and E46K or Alzheimer’s-related proteins Aβ and the K18 fragment of Tau were detected by TCCD and the equilibrium concentrations of co-oligomers produced under a wide range of initial monomer concentrations were analyzed using a statistical mechanical model [116]. From this, the free energies of oligomer formation were obtained, showing that co-oligomer formation is more favorable than self-oligomer formation. Using an ultrasensitive liposome assay based on single-molecule TIRF imaging, in which the disruption of the membrane and the influx of calcium ions is reflected in the fluorescence enhancement of a calcium ion binding dye in the liposome [117], the membrane permeabilization by the amyloid oligomers can be quantified and related to the toxicity. It was shown that although self-oligomers have higher ability to disrupt lipid membranes, the formation of co-oligomers is more favorable than formation of self-oligomers, counteracting the difference in toxicity between self- and co-oligomers, which highlights the important role of co-oligomers in neurodegenerative diseases [116].

### 2.5. The Effects of Different Factors on the Amyloid Aggregation Pathway

The establishment of single molecule fluorescence techniques has greatly facilitated the study of the mechanisms of amyloid oligomerization and fibrillization and shed light on the relationship between oligomer properties and cytotoxicity. In this way, how different factors such as molecular chaperones, antibodies, short peptides, small molecules and nanomaterials modify the pathway of amyloid formation can also be revealed in detail (Figure 3C and Table 1), which not only helps us to understand the molecular mechanism but also provides a theoretical basis for development of therapeutic strategies for neurodegenerative diseases. Extensive studies on inhibition mechanisms of amyloid fibril formation in ensemble systems have been reviewed elsewhere [118,119,120], whereas here the focus will be on single molecule studies.

Chaperones play a key role in preventing protein misfolding and aggregation, thereby protecting cells from a variety of protein misfolding diseases [121,122,123]. Heat shock protein 70 (Hsp70) is a key chaperone in maintaining protein homeostasis and inhibiting protein misfolding and aggregation. As an important regulator in the pathogenesis of Alzheimer’s disease and other tauopathies, it has been found that Hsp70 can inhibit the fibril formation of Tau [124,125]. The inhibition mechanism of human Hsp70 on amyloid aggregation of the K18 fragment of Tau with the ΔK280 mutation was explored using smFRET [126]. Hsp70 binds and stabilizes monomeric and small oligomeric species of Tau and inhibits the primary nucleation. Moreover, Hsp70 sequesters growth competent seeds and inhibits their elongation by monomer addition. The affinity of Hsp70 increases with the size of aggregates of K18. These findings suggest that Hsp70 may efficiently neutralize the ability of the oligomeric species to damage membranes.

The inhibition of amyloid aggregation of Aβ by intracellular and extracellular chaperones has been studied using smFRET and TCCD. The oligomeric intermediates formed by Aβ40 show a heterogeneous size distribution [62]. The kinetics and thermodynamics of oligomer formation during the aggregation reaction or released from mature fibrils during disaggregation were determined. In the presence of the extracellular chaperone clusterin, Aβ40 oligomers from both aggregation and disaggregation processes can interact with clusterin to form long-lived, stable complexes [62]. Using the same method, the intracellular chaperone, αB-crystallin, was also found to bind and stabilize misfolded oligomeric species, thereby preventing their further growth into fibrils as well as their dissociation [127], suggesting a common protective role of these chaperones. Using a variety of biophysical methods including solution NMR, EM, atomic force microscopy (AFM), DLS, and smFRET, Wälti et al. [128] showed that transient interactions of GroEL with Aβ42 monomers slow down the rate of appearance of protofibrils and fibrils, providing a mechanistic basis for the neuroprotective properties of heat shock protein 60 (Hsp60).

The effect of chaperones has also been explored in other amyloid proteins, such as α-synuclein. With FRET pair labeling on the chaperone and α-synuclein, Whiten et al. [129] found that the extracellular chaperones clusterin and α2-macroglobulin can directly bind to α-synuclein oligomers mediated by hydrophobic interactions. The binding of either chaperone reduces the ability of the oligomers to permeabilize membranes and reduces reactive oxygen species (ROS) induction in neuronal cells. Small heat shock protein Hsp27 can also inhibit the fibril formation of α-synuclein. Using single aggregate visualization by enhancement (SAVE) imaging [64,130], Cox et al. [130] showed that Hsp27 binds to α-synuclein fibrils along the surface and thus decreases the hydrophobicity and the cellular toxicity of fibrillar α-synuclein. These findings taken together indicate that sequestering both oligomeric and fibrillar species formed during amyloid aggregation is a common working mode of chaperones to decrease the hydrophobicity and toxicity, inhibit the generation, evolution and spreading of toxic oligomers, stabilize the oligomers and seeds and disturb processes including primary nucleation, elongation, dissociation and secondary nucleation, which consequently reduces the damage to the cell membrane.

In addition to naturally occurring molecular chaperones, various kinds of aggregation inhibitors have been designed and investigated [120,131,132,133]. Inhibition strategies have focused on several aspects [134,135,136,137]: (1) sequestering/blocking of the monomers and aggregation products including intermediates and protofibrils; (2) promoting the degradation of preformed oligomeric species; (3) altering the aggregation processes to reduce the accumulation of toxic species by slowing down the aggregation process. In an earlier study, Caruana et al. [138] took advantage of FCS, fluorescence-intensity distribution analysis (FIDA) and scanning for intensely fluorescent targets (SIFT) techniques to study the effects of 14 naturally-occurring polyphenolic compounds and black tea extract on α-synuclein oligomer formation, and found polyphenols show potential inhibition by disaggregating the pre-formed oligomers. Arginine and glutamate have been reported to influence the competition between conformational fluctuations and oligomerization of α-synuclein differently, with arginine acting as an inhibitor that shifts early species towards a compact conformation while glutamate acts as a facilitator that speeds up oligomer formation, as revealed by FCS [139]. Another example of effective inhibitors are single domain antibodies (nanobodies), which have been reported to inhibit the fibril formation of α-synuclein [140,141,142]. SmFRET was used to detect changes in the two types of α-synuclein oligomers with low- or high-FRET efficiencies with or without the nanobodies, which reveals that binding of nanobodies to α-synuclein accelerates the reverse conversion from stable oligomer (high-FRET) to less stable oligomer (low-FRET), leading to reduction of cytotoxicity [107].

In addition to inhibitors, some accelerators of fibril formation can also decrease cytotoxicity [143,144], although the mechanism of this still needs detailed investigation. Recently, we identified a short peptide (LQVNIGNR) derived from the yeast prion protein Ure2 that is capable of forming vesicular structures and accelerates fibril formation of both Ure2 and other unrelated proteins including Tau and α-synuclein [145]. By combined smFRET and kinetic analysis, we found that the peptide vesicles promote the conformational conversion of oligomeric intermediates of Ure2 into fibrillar species in a catalytic manner. In contrast to the common strategy of short peptides as amyloid inhibitors [131], this work suggests an alternative strategy for reducing the cellular toxicity caused by amyloid aggregation via accelerating amyloid formation and so reducing the time period during which toxic oligomeric intermediates are present.

**Table 1:** The effects of different factors on amyloid aggregation revealed by single molecule study.

Factor [with Reference]	Amyloid Protein	Effect on Fibril Formation	Microscopic Mechanism
Heat shock protein 70 (Hsp70) [126]	Tau	Inhibition	Stabilizes monomer and oligomer; sequesters fibril seeds
Clusterin [62]	Aβ40	Inhibition	Interacts with oligomers to form stable complexes
αB-crystallin [127]	Aβ40	Inhibition	Stabilize oligomers to prevent their growth into fibrils
GroEL [128]	Aβ42	Inhibition	Interacts with monomers
Clusterin [129]	α-synuclein	Inhibition	Binds to oligomers
α2-macroglobulin [129]	α-synuclein	Inhibition	Binds to oligomers
Heat shock protein (Hsp27) [130]	α-synuclein	Inhibition	Binds to the fibrillar species to inhibit elongation
Polyphenols [138]	α-synuclein	Inhibition	Disaggregates preformed oligomers
Arginine [139]	α-synuclein	Inhibition	Changes oligomer conformations
Glutamine [139]	α-synuclein	Acceleration	Promotes oligomer formation
Nanobodies [107]	α-synuclein	Inhibition	Changes oligomer conformations
Short peptide [145]	Ure2	Acceleration	Promotes oligomer conversion

## 3. Conclusions

Over the past decade, with the development of ultrasensitive single molecule fluorescence techniques, amyloid oligomers have been thoroughly and quantitatively characterized, despite their low-populated, dynamic and heterogenous nature. The thermodynamic and kinetic properties of amyloid oligomers measured in vitro can be extrapolated to physiological conditions that are not accessible experimentally. This progress has greatly improved our understanding of the mechanism of amyloid aggregation and its relationship with neurodegenerative diseases. To date, the majority of single molecule studies on amyloid aggregation have been performed in vitro, but the simplified experimental conditions in vitro may not fully represent the more complicated cellular context. Due to the difficulty in achieving fluorescence labeling as well as improving the signal to noise ratio and fluorescence stability in vivo, it remains challenging to apply single molecule detection to living cells. However, a promising approach is the introduction of dye labels into living cells with the recent applications of microinjection or electroporation [146,147]. Meanwhile, advanced illumination techniques such as light-sheet or single-plane illumination microscopy that enable 3D sectioning with highly reduced background and limited phototoxicity provide opportunities for in vivo single molecule measurements of protein dynamics [148]. These and anticipated further development of new techniques will no doubt facilitate in vivo studies of the assembly of amyloid proteins at the single molecule level, therefore providing guidance on potential therapies for neurodegenerative diseases in the future.
